# Curcumin and Omega-3 Fatty Acids Enhance NK Cell-Induced Apoptosis of Pancreatic Cancer Cells but Curcumin Inhibits Interferon-γ Production: Benefits of Omega-3 with Curcumin against Cancer

**DOI:** 10.3390/molecules20023020

**Published:** 2015-02-12

**Authors:** Milan Fiala

**Affiliations:** Department of Surgery, School of Medicine, University of California, Los Angeles, CA 90095, USA; E-Mail: Fiala@mednet.ucla.edu; Tel.: +1-310-206-6392

**Keywords:** natural killer cells, curcuminoids, omega-3 fatty acids, pancreatic cancer

## Abstract

STAT-3 and STAT-1 signaling have opposite effects in oncogenesis with STAT-3 acting as an oncogene and STAT-1 exerting anti-oncogenic activities through interferon-γ and interferon-α. The cytokine IL-6 promotes oncogenesis by stimulation of NFκB and STAT-3 signaling. Curcuminoids have bi-functional effects by blocking NFκB anti-apoptotic signaling but also blocking anti-oncogenic STAT-1 signaling and interferon-γ production. In our recent study (unpublished work [1]) in pancreatic cancer cell cultures, curcuminoids enhanced cancer cell apoptosis both directly and by potentiating natural killer (NK) cell cytotoxic function. The cytotoxic effects of curcuminoids were increased by incubation of cancer cells and NK cells in an emulsion with omega-3 fatty acids and antioxidants (Smartfish), which enhanced cancer cell apoptosis and protected NK cells against degradation. However, as also shown by others, curcuminoids blocked interferon-γ production by NK cells. The combined use of curcuminoids and omega-3 in cancer immunotherapy will require deeper understanding of their *in vivo* interactions with the immune system.

## 1. Inflammatory Signaling by NFκB in Cancer

Curcuminoids have a significant potential in the therapy of tumors with inflammatory mechanisms through their activity as potent inhibitors of the transcription factors NFκB and Signal transducer and activator of transcription 3 (STAT-3) and their downstream targets [2]. However, curcuminoids also inhibit STAT-1 signaling necessary for anti-tumor responses through interferon-γ (IFN-γ). Constitutive activity of NFkB is found in the haematological malignancies multiple myeloma, lymphomas, myelodysplastic syndrome and leukemias, and most solid tumors. In natural killer (NK) T cell lymphoma, NFκB is constitutively active and is blocked by curcumin, which induces apoptosis of cancer cells by down regulation of the anti-apoptotic genes induced by NFκB: *BCLXL* (also known as *BCL2L1*), cyclin D1 (*CCND1*), X-linked inhibitor of apoptosis protein (*XIAP*) and c-FLIP [3,4]. As discussed below, by inhibiting NFκB, curcumin has positive anti-tumor effects by stimulating apoptosis of cancer cells but possibly negative effects on anti-tumor immunity by suppression of IFN-γ. The importance of curcumin in cancer therapy dependent on the immune effects of natural killer (NK) cells is significantly potentiated by combination with omega-3 fatty acids as shown in our recently submitted manuscript [1]. Indeed, the synergistic effects of curcumin and omega-3 fatty acids are appreciated in breast cancer models where this combination treatment reduced breast tumor incidence [5], and in pancreatic cancer xenograft model where it reduced the tumor volume [6]. However, the augmentation of NK cell cytocidal activity by curcumin and omega-3 fatty acids has not been published before the results reported in our manuscript under consideration.

## 2. Immune Control and Tumor Escape

Although initially the immune cells eliminate cancer cells, they ultimately fail and actually promote oncogenesis actively through IL-6 [7]. IL-6 activates STAT3 constitutively both in tumor cells and immune cells. The oncogenic effects of STAT3 are mediated through anti-apoptotic effects by upregulation of BCL-X [8]. Persistently activated STAT3 in cancer cells also leads to suppression of the anti-oncogenic cytokines interferons Type I (α and β) and Type II (γ) [9]. Curcumin markedly inhibits the phosphorylation of STAT1 and STAT3 as well as JAK1 and JAK2 through activation of Src Homology 2 Domain-Containing Tyrosine phosphatase (SHP-2), thus contributing to suppression of IFN-γ signaling [10]. Curcumin also attenuates phosphorylation of the transcription factor STAT5 [11], which up regulates NK cell-mediated cytolytic activity [12].

## 3. *In Vivo* Anti-Oncogenic Effects of Curcumin

Curcumin in combination with gemcitabine therapy had promising results in pancreatic cancer patients [13]. The demonstration of cytostatic and cytotoxic effects against tumors of multiple origins in cell culture and animal models and a lack of immunosuppressive properties in animal models stimulated optimistic reports for curcumin potential in cancer therapy (Table 1). In animals given curcumin by intraperitoneal injection, the investigators found no interference with the cytotoxic function of NK cells, the generation of reactive oxygen species and nitric oxide from macrophages, and the production of Th1 regulatory cytokines [14]. However, curcumin decreased nitric oxide production during the induction of antitumor responses by IL-2 in a mouse ascites tumor model [15]. Recent studies show *in vivo* effects of curcumin on orthotopic pancreatic cancer mouse model [16] and isolated effects in patients with pancreatic cancer [17]. In addition, curcumin has benefits as an enhancer of radiation and chemotherapy therapies and a protector of normal tissues [18] (Table 2). To improve the anti-oncogenic effects, new synthetic curcuminoids have been synthesized containing piperidone [19], and other analogues, such as CDF [20], and other patented analogues [21] have been published. Improved delivery of curcuminoids in liposomes and nanoparticles and longer circulation time of difluoro analogues are of current interest [22] and are relevant for administration of curcuminoids to cancer patients in omega-3 emulsion [1].

**Table 1:** Curcuminoid effects against pancreatic cancer in rodent models and clinical trials.

Authors	Model	Results of Curcumin	Reference
Bimonte, S. *et al.* (2013)	Orthotopic mouse model with MP2 cells injected into pancreas of nude mice	Smaller tumors, down regulation of NF-κB	[16]
Dhillon, N. *et al.* (2008)	Phase ll in advanced pancreatic cancer	Curcumin blood level (22–41 ng/mL); stimulation of IL-6 in the blood; clinical effects in 2 patients	[17]
Goel, A. and Aggarwal, B.B. (2010)	Rodent models	Chemosensitiser(e.g., doxorubicin) and radiosensitiser and protector of tissue	[18]

**Table 2:** New and synthetic curcumin-related compounds.

Author	Name	IC_50_	Reference
Zhou, D.Y. *et al.* (2013)	Benzyl piperidone	<2 μM Inhibition of pancreatic cancer cell lines	[19]
Wei, X. *et al.* (2012)	61 New synthetic curcuminoids	<1 μM	[21]
Dandawate, P. R. *et al.* (2012)	CDF (analogue of curcumin) in complex with β-cyclodextrin	50% Reduction in IC50; CDF inhibits tumor growth and modulates metastatic spread by regulating miR-21 and miR-200 expression	[20]
Padhye, S. *et al.* (2010)	Curcumin analogues (difluoro.)	Delivery systems as liposomes, phospholipid complexes, and nanoparticles	[22]

## 4. Anti-Oncogenic Effects of Curcumin in Cell Culture

The pro-apoptotic and anti-inflammatory effects of curcumin were confirmed in human melanoma cell cultures. However, a major caveat was found in curcumin blockade of phosphorylation of STAT1 and STAT3 in melanoma cells and peripheral blood mononuclear cells (PBMCs) and inhibition of the principal anti-tumor cytokine IFN-γ production by NK cells [11]. Curcumin increased in a dose-dependent fashion apoptosis of human melanoma cell lines, which was most prominent at doses >10 µmol/L. However, curcumin also inhibited the ability of IFN-α, IFN-γ, and IL-2 to phosphorylate STAT1 and STAT5, critical for their antitumor activity, and their respective downstream gene expression. By blocking phosphorylation of STAT-1 and STAT-3 as well as NFκB signaling, curcumin had bi-functional effects on cancer: promotion of apoptosis but blockade of the principal anti-oncogenic cytokine IFN-γ (Type II interferon). These effects of curcumin should be compared to the effects of IFN-α (Type I interferon), a time-honored therapy of melanoma. IFN-α modulates the balance of pSTAT1/pSTAT3 in melanoma cells and lymphocytes and the increased baseline ratio is a predictor of therapeutic effect [23]. A potential curcumin-based approach that does not reduce IFN-γ but is an inhibitor of STAT-3 pathway is through a small molecule FLLL32 derived from curcumin [24].

## 5. Omega-3 Fatty Acids in Cancer Prevention

Recently, attention to nutritional prevention of cancer has focused on altering the diet to increase omega-3 fatty acids (docosahexaenoic acid (DHA) and eicosapentaenoic acid (EPA)) and to decrease the omega-6 fatty acid arachidonic acid. The diet rich in marine omega-3 fatty acids decreases pro-inflammatory eicosanoids (prostanoids and leukotrienes) and increases “Special pro-resolving mediators” (SPMs) (resolvins, protectins and maresins) [25]. It was shown that the decreased ratio of omega-6/omega-3 (in fat-1 mouse model with a balanced n-6/n-3 production) attenuates cancer growth though up regulation of PTEN pathway mediated by prostaglandin E3 (PGE3) [26]. A broad anti-oncogenic approach has been proposed to include eicosanoid receptor antagonism, overexpression of eicosanoid metabolizing enzymes, and the use of endogenous SPMs [27].

## 6. Combined *in Vitro* Effects of Omega-3 and Curcumin against Cancer Cells

To discover a practical combination approach to immunotherapy by curcuminoids and omega-3 fatty acids, we have tested both direct effects of curcuminoids plus omega-3 with antioxidants (Smartfish) and combined effects of curcuminoids, omega-3 with anti-oxidants plus NK cells on the MP2 pancreatic cancer cells. The results [1] showed that curcuminoids (10 microM > 1 microM) cause apoptosis of pancreatic cancer cells when the cancer cells are incubated in a cell-culture medium with an emulsion of omega-3 and antioxidants (Smartfish) but not in a commercial fish-oil (from sardines). In addition, curcuminoids increased cytotoxic activity of NK cells but only when incubated in the medium with an emulsion of omega-3 and antioxidants. However, our results in pancreatic cancer cells again showed blockade of IFN-γ production in NK cells by curcumin. In two different experiments, curcumin decreased IFN-γ production by ~75% and 50% respectively. Interestingly, in NK cells from a patient with disseminated prostate cancer, the lipidic mediator resolvin D1 [28] actually increased IFN-γ production in vitro. Importantly, we found that omega-3 with anti-oxidants (Smartfish) stimulated NK cells from different donors to increase their pancreatic cancer cell cytotoxic effects by >100%. In addition, microscopic examination showed that a combination of omega-3 with anti-oxidants (Smartfish) in cell culture medium protects NK cells against degradation after overnight incubation with cancer cells.

## 7.* In Vivo* Effects of Omega-3 with Antioxidants on NK Cells

NK cells become inactivated during chemotherapy and are less active than normal donors’ NK cells [29]. We have recently had a surprising single-patient experience with a nutritional supplementation by the Smartfish drink (omega-3 with anti-oxidants and curcuminoids) (Smartfish, Oslo, Norway) of a patient with metastatic prostate cancer who received supplementation with the drink daily for 3 months. His NK cells had higher cytotoxic activity than NK cells of normal donors who had no supplementation [1].

## 8. Coda

NK cells express a repertoire of activating and inhibitory receptors that recognize either lack of MHC class I expression or overexpression of NKG2D ligands on tumor cells [30]. Immune surveillance by NK cells and therapeutic effects of NK cells against tumors are exciting strategies, but tumor cells deactivate NK cells by activation-induced cell death. The combined effects of three different small molecules, omega-3 fatty acids (DHA and EPA), resolvin D1 derived *in vivo* from omega-3, and curcuminoids have a potential to significantly change the balance of the battle between tumor and NK cells. Omega-3 fatty acids with anti-oxidants protect NK cells in their interaction with tumor cells. Curcuminoids have bi-functional tumor effects, positive pro-apoptotic and anti-IFN-γ effects, which are considered two-faced against cancer [31]. Omega-3 act through different specialized proresolving mediators (SPMs), resolvins, protectins [28] and maresins [32], attenuate inflammation, stimulate resolution and potentially increase anti-tumor defenses. SPMs affect a variety of cells, including endothelial cells, platelets and immune cells. Unfortunately, in case of patients with gastrointestinal malignancies, there is no clear understanding of the distribution of these molecules and their receptors, and their spatial and temporal interactions with the tumor cells, NK, macrophages, dendritic cells and other immune cells. Future *in vivo* studies need to analyze the interactions and indicate a direction for clinical application of these exciting *in vitro* observations.
